# Supplementary material for: Synthesis, Electronic Structure, and Electrochemical Properties of the Cubic Mg2MnO4 Spinel with Porous-Spongy Structure
Source: Nanomaterials (Basel). 2021 Apr 27;11(5):1122. doi: 10.3390/nano11051122 (PMC8145627; doi:10.3390/nano11051122)
Supplement: Supplementary file 1 [file nanomaterials-11-01122-s001.zip › nanomaterials-1183736-supplementary.pdf]

nanomaterials

MDPI

Article

Synthesis, Electronic Structure, and Electrochemical Properties of the Cubic Mg_2MnO_4 Spinel with Porous-Spongy Structure

Zhenyan Wang [1], He Zhu [1], Li Ai [1], Jimin Ding [1], Pengfei Zhu [1], Ziqing Li [1], Bo Li [1], Hechun Jiang [1], Fapeng Yu [1], Xiulan Duan [1,*] and Huaidong Jiang [1,2]

[1] State Key Laboratory of Crystal Materials, Institute of Crystal Materials, Shandong University, Jinan 250100, China; 18839199546@163.com (Z.W.); zhuhe@mail.sdu.edu.cn (H.Z.); 201912593@mail.sdu.edu.cn (L.A.); jiminding@mail.sdu.edu.cn (J.D.); joseph.zhu.nt@gmail.com (P.Z.); lzq@fudan.edu.cn (Z.L.); boli@sdu.edu.cn (B.L.); jianghechun001@126.com (H.J.); fapengyu@sdu.edu.cn (F.Y.); jianghd@shanghaitech.edu.cn (H.J.)

[2] School of Physical Science and Technology, ShanghaiTech University, Shanghai 201210, China

* Correspondence: xlduan@sdu.edu.cn

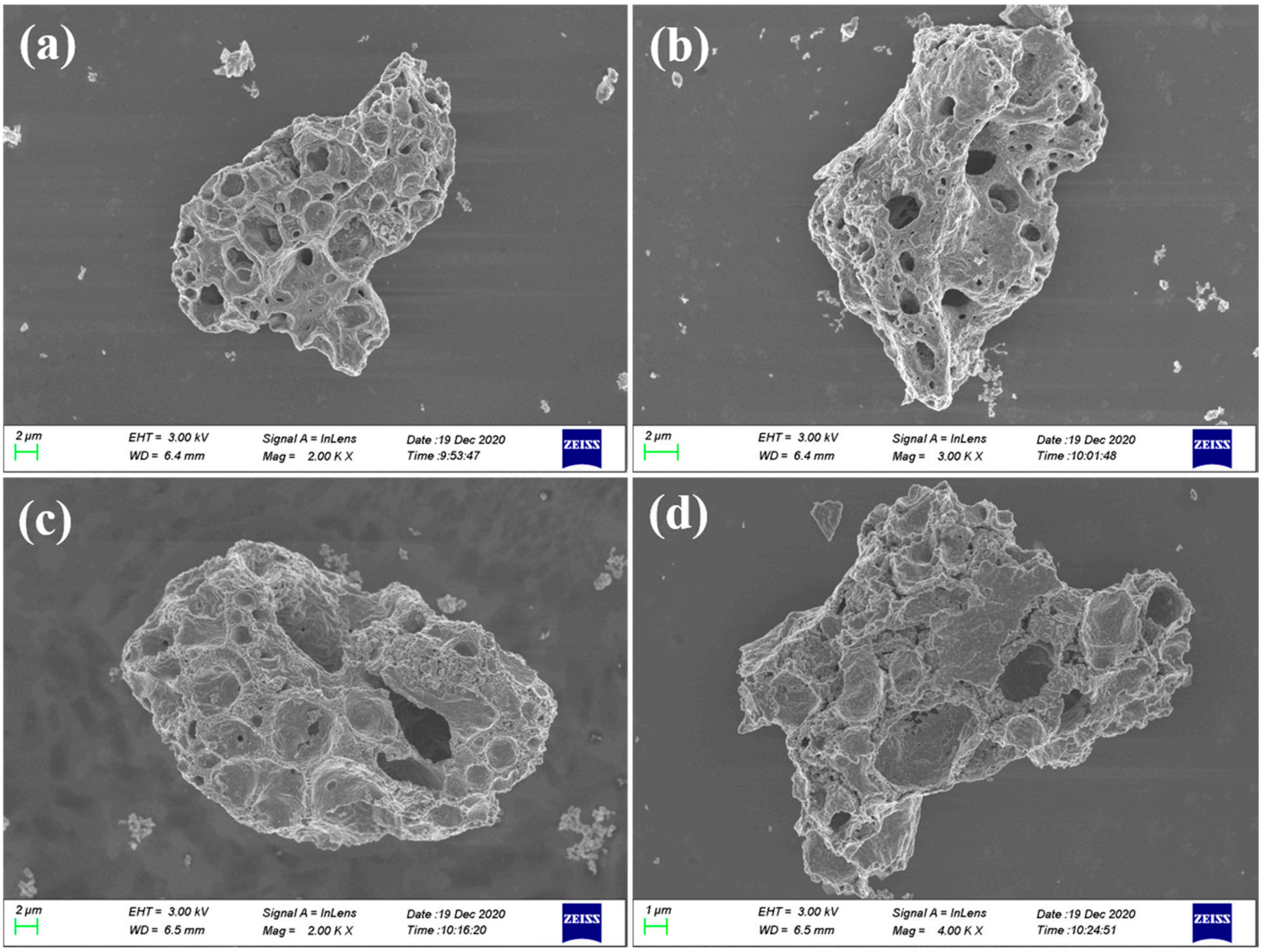

Figure S1. SEM images of Mg_2MnO_4 nanoparticles annealed at different temperatures: (**a**) 500 °C, (**b**) 600 °C, (**c**) 700 °C, (**d**) 800 °C.

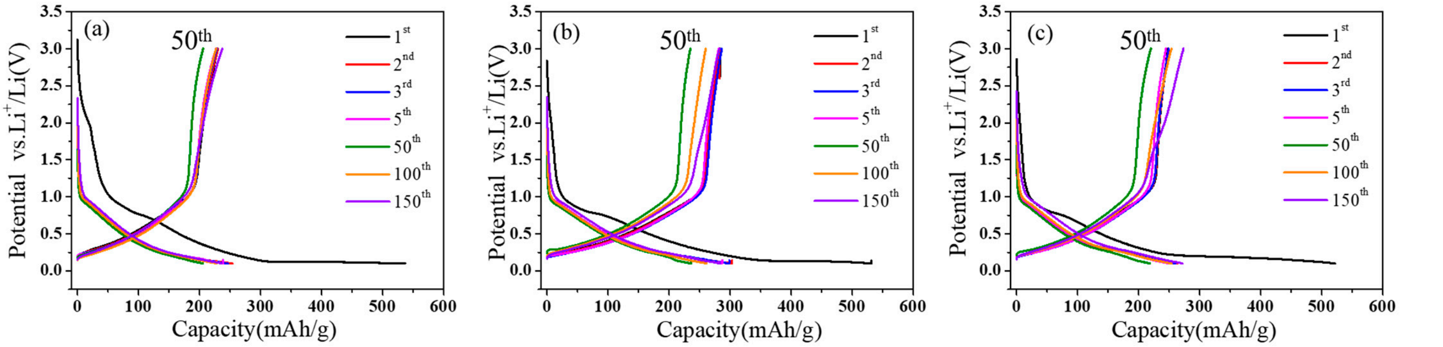

Figure S2. The 1, 2, 3, 50, 100 and 150 cycles of charge/discharge curves at a rate of 1C. (**a**) 500 °C; (**b**) 700 °C; (**c**) 800 °C.

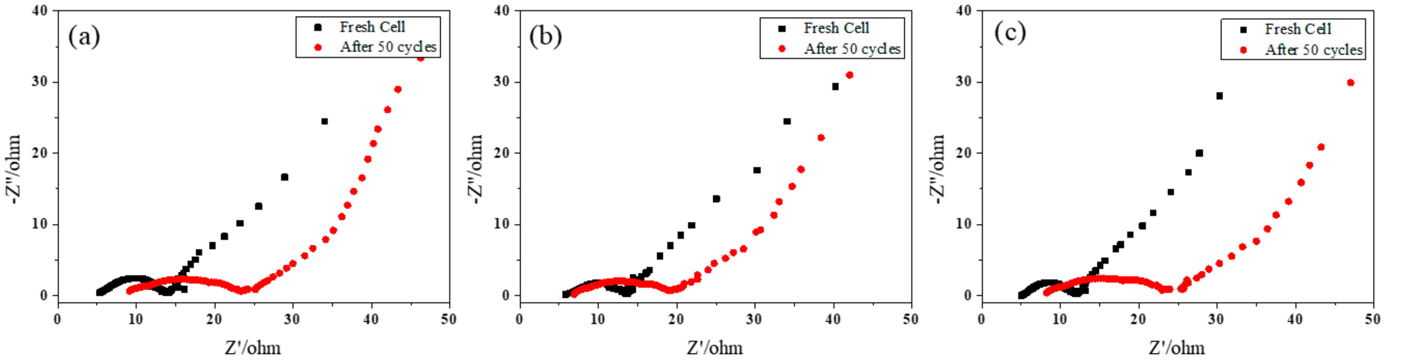

Figure S3. Nyquist plots of the electrode of Mg_2MnO_4 operating at the fresh cell and after 50 cycles. (**a**) 500 °C; (**b**) 700 °C; (**c**) 800 °C.

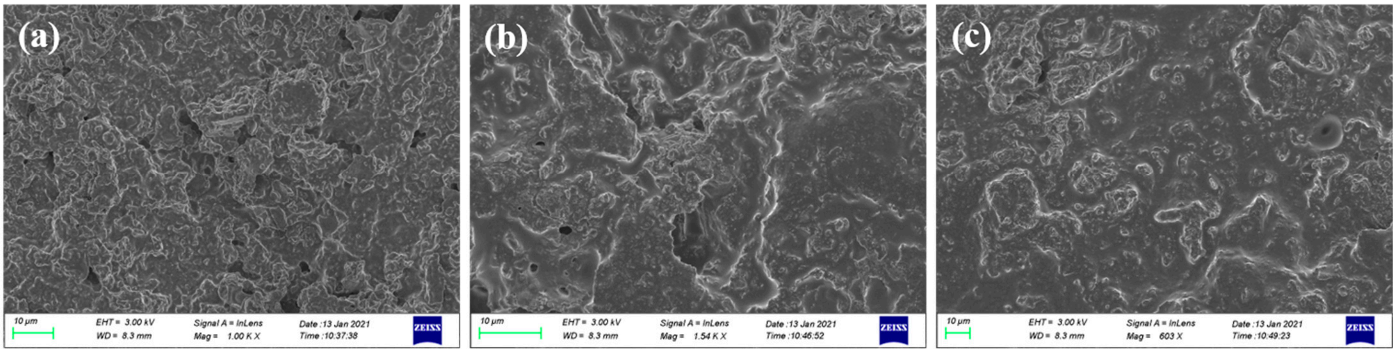

Figure S4. The SEM image of the anode material after 50 cycles. (**a**) 500 °C; (**b**) 700 °C; (**c**) 800 °C.

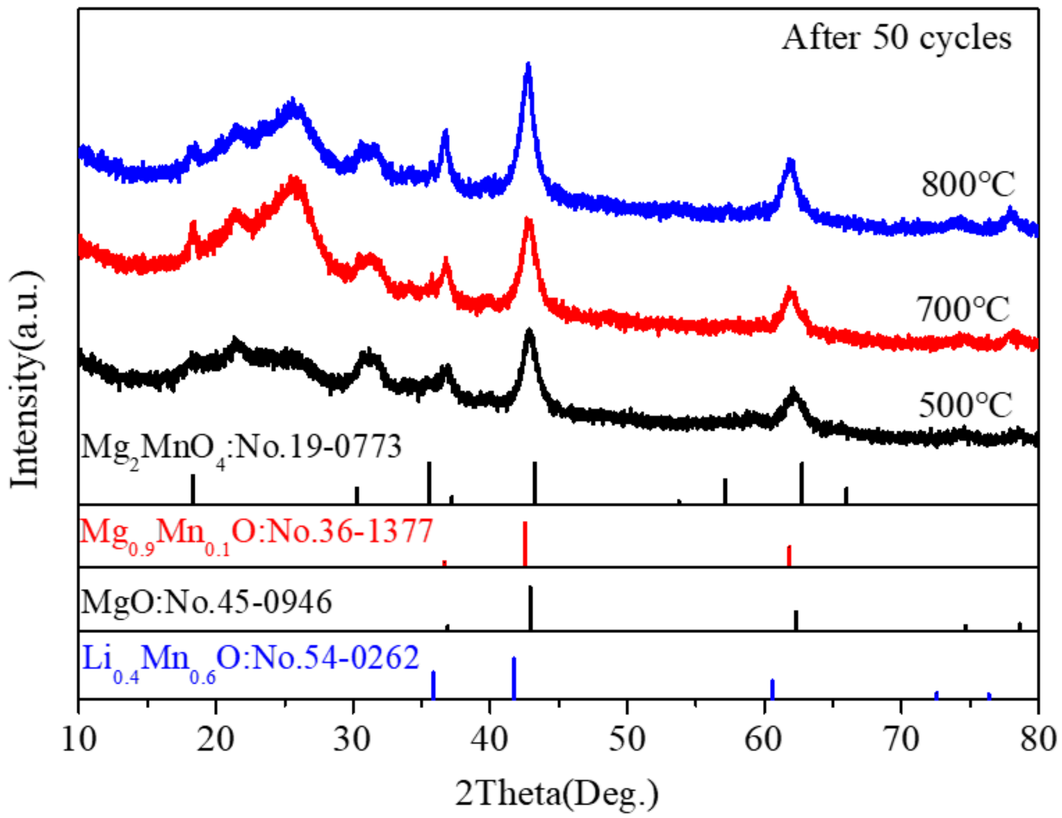

Figure S5. The XRD patterns of the material after 50 cycles at different annealed temperature.
